# Low Ang-(1–7) and high des-Arg9 bradykinin serum levels are correlated with cardiovascular risk factors in patients with COVID-19

**DOI:** 10.1515/med-2023-0741

**Published:** 2023-07-04

**Authors:** Farzaneh Rostamzadeh, Hamid Najafipour, Samira Nakhaei, Rostam Yazdani, Ahmad Alinaghi Langari

**Affiliations:** Physiology Research Center, Institute of Neuropharmacology, Kerman University of Medical Sciences, Kerman, Iran; Cardiovascular and Respiratory Physiology, Cardiovascular Research Center, Institute of Basic and Clinical Physiology Sciences, Kerman University of Medical Sciences, Kerman, Iran; Endocrinology and Metabolism Research Center, Institute of Basic and Clinical Physiology Sciences, Kerman University of Medical Sciences, Kerman, Iran; Department of Internal Medicine, School of Medicine, Afzalipour Hospital, Kerman University of Medical Sciences, Kerman, Iran; Gastroenterology, and Hepatology Research Center, Institute of Basic and Clinical Physiology Sciences, Kerman University of Medical Sciences, Kerman, Iran

**Keywords:** angiotensin-(1–7), des-Arg (9)-bradykinin, COVID-19, cardiovascular risk factors

## Abstract

It is predictable that the renin–angiotensin–aldosterone and kinin–kallikrein systems are dysregulated in COVID-19 (COV) patients because SARS-CoV-2 requires ACE2 to cause an infection. This study aimed to assess the serum levels of des-arg(9)-bradykinin (DABK) and angiotensin 1–7 (ang-(1–7)) in patients with COV who had the above-mentioned cardiovascular disease risk factors. In a cross-sectional study, 69 COV patients were selected among patients referred to the main referral center for these patients, in Kerman, Iran, and 73 matched control (non-COV) individuals among individuals who participated in the KERCARD cohort study. Serum levels of DABK and ang-(1–7) were measured by ELISA in the groups of CTL (healthy), HTN, DM, OB, COV, COV + HTN, COV + DM, and COV + OB. Ang-(1–7) levels were lower in the COV + HTN group compared to the HTN group. DABK levels were higher in the COV, HTN, and OB groups and in DM + COV subjects compared to their corresponding control group. The levels of ang-(1–7) and DABK were related to HTN and OB, respectively. According to the findings, we can infer that an increase in DABK production in those with the cardiovascular disease risk factors of diabetes, obesity, and hypertension or a decrease in ang-(1–7) in those with hypertension may contribute to the adverse outcomes of SARS-CoV-2 infection.

## 1 Introduction

Since the onset of the COVID-19 (COV) pandemic in Wuhan, China, in November 2019, millions of people have suffered from this disease worldwide, and this infection is still circulating across the world [1]. Patients with COV suffer from acute respiratory distress syndrome (ARDS), inflammation, and deleterious secondary effects, such as lung fibrosis, cardiovascular disease, and diabetes [2,3].

As the COV virus needs angiotensin-converting enzyme-type 2 (ACE2), which is one of the members of the renin–angiotensin–aldosterone system (RAAS), to infect cells, the virus possibly changes the activity and levels of ACE2 and disturbs RAAS stability [4]. Indeed, clinical findings in COV patients and experimental findings in mice that were infected with spikes of severe acute respiratory syndrome coronavirus 2 (SARS-CoV-2) have indicated RAAS system instability [3,5,6]. The increase in angiotensin II (ang-2) production is associated with severe symptoms and detrimental outcomes such as inflammation, respiratory distress syndrome, and thrombolytic complications [3].

Angiotensin 1–7 (ang-(1–7)), which is one of the products of ACE2 activity, ameliorates the classic effects of RAAS activation, including vasoconstriction, inflammation, oxidative stress, and fibrosis through MAS and AT2 (angiotensin type 2) receptors [7]. The ang-(1–7)/MAS-R signaling pathway is beneficial to several pulmonary diseases, including SARS-CoV-1-mediated lung injuries [8,9].

ACE2 also preserves the stability of the kinin–kallikrein system (KKS) by converting the active form of the bradykinin metabolite, des-arg(9)-bradykinin (DABK) to its inactive metabolites [10]. Extra accumulation of DABK in tissues activates bradykinin receptor B1. Activation of this receptor increases the rate of fluid leakage and mobilization of leukocytes in tissues [11].

According to previous knowledge, KKS regulation is anticipated to be interrupted in COV patients. It has also been established that the RAAS and KKS are dysregulated in hypertension, diabetes, and obesity [12,13,14]. Furthermore, evidence suggests that people with pre-existing comorbidities such as cardiovascular disease, hypertension, diabetes, and obesity are more prone to severe symptoms of COV and have a higher mortality rate [15,16,17]. SARS-CoV-2 binding to ACE2 at the vascular endothelium leads to endothelial dysfunction by increasing oxidative stress and/or inflammatory responses [18]. These alter the production of endothelium-related products such as ang-(1–7) [19]. Disturbances in the balance between ang-(1–7) and ang-2 cause damage to the vascular endothelium. In the previous study, it was observed that the changes in apelin levels, another substrate of ACE2, were associated with arterial O_2_ saturation, hospitalization period, and degree of lung involvement and pre-existing comorbidities, such as diabetes mellitus, obesity, and hypertension in COVID patients [20]. In this study, we aimed to assess the levels of ang-(1–7) and DABK in COV patients with the abovementioned pre-existing comorbidities in comparison with their non-COV counterparts.

## 2 Materials and methods

All procedures performed in this study involving human participants were in accordance with the ethical standards of the Declaration of Helsinki. The Ethics Committee of Kerman University of Medical Sciences approved the experimental protocol (Ethic code: IR.KMU.REC.1399.586). The Kerman coronary artery disease risk factors (KERCADR) study that the control groups were selected from, had its own approval (Ethic code: IR.KMU.REC.1393.310). The participants signed a written informed consent form.

The enzyme-linked immunosorbent assay (ELISA) kits of ang-(1–7) and DABK were purchased from Bioassay Technology Laboratory (China) and MyBioSource, Inc (San Diego, USA), respectively.

### 2.1 Subjects and sampling

In total, 144 subjects were enrolled in this cross-sectional study. Sixty-nine COV patients were selected from patients referred to Afzalipour Hospital in Kerman, Iran, the main referral center in the city, from February to November 2020. Seventy-three individuals who took part in KERCADRS, Phase 3 during the same timespan was chosen and matched as the control group [21]. We did our best for the corresponding control cases to be matched with COV cases in terms of gender, age, and BMI. The follow-up period was 1 to 33 days with a mean of 10.3 days from hospitalization to discharge.

Reverse transcription-polymerase chain reaction (RT-PCR) was used to confirm COV infection. The severity of COV infection was almost identical in test subjects on the first day of hospitalization when the blood samples were taken. The blood samples were centrifuged at 4,000*g*, and serum samples were separated and kept at −80°C. The serum levels of ang-(1–7) and DABK were determined using ELISA kits according to the manufacturer’s protocols. The study groups were as follows:

(1) Control (CTL, *n* = 20): healthy individuals who did not have COV or any pre-existing risk factors, (2) HTN: hypertensive (*n* = 20), (3) DM: diabetic (*n* = 18), (4) OB: obese (*n* = 15), (5) COV (COV-19) (*n* = 20): patients with COV who had no pre-existing risk factor, (6) COV + HTN (*n* = 18), (7) COV + DM (*n* = 15), and (8) COV + OB (*n* = 18). The number of subjects in the groups was chosen according to the following formula [22] with a 5% type 1 error, which is 1.96, and a power of 0.8, which is 0.84. The mean and standard deviation were chosen from a previous study reporting the ang-(1–7) level in normal and gestational hypertensive subjects [23]:

{\rm{Sample\; size}}=\left(\frac{r+1}{1}\right)\left(\frac{{{SD}}^{2}{\left(Z\beta +\frac{Z\alpha }{2}\right)}^{2}}{{d}^{2}}\right)\left=\left((1\left+1)\left/1)\left(\frac{{11}^{2}{(0.89+1.96)}^{2}}{{8}^{2}}\right)=15.3.]

The hypertensive participants had a systolic blood pressure ≥140 mmHg or a diastolic blood pressure ≥90 mmHg or took antihypertensive medications [24]. Participants with diabetes were chosen from those with a fasting blood sugar level ≥126 mg/dL or who were receiving anti-diabetic medications [25]. Obesity is defined as a body mass index (BMI) ≥ 30 kg/m^2^ [26]. Since COV increases the risk of hyperglycemia and hypertension [27], COV participants with diabetes and hypertension were selected based on their history of having these risk factors.

### 2.2 Statistical analysis

The data were analyzed by using SPSS Statistics Version 26. Data in the tables and figures are presented as mean ± SEM for continuous data and as the frequency for categorical variables. After checking for normality in data distribution, two-way ANOVA was performed. The two independent factors were pre-existing disease (with four variables: control, hypertension, diabetes, and obesity) and COV infection (with two categories: yes/no). Differences between continuous variables were analyzed using one-way ANOVA followed by Tukey’s *post hoc* test. The categorical variables were analyzed using the chi-square test. A linear regression test was used to assess the association between ang-(1–7) and DABK levels with risk factors. Multivariable linear regression was used to assess the predictors of ang-(1–7) and DABK levels in combined groups. The models were adjusted for gender, age, and obesity. Cox regression was performed to evaluate the hazard of death related to different variables. *P*-values < 0.05 were considered significant.

## 3 Results

The general features of the studied groups are shown in Table 1. The age and gender of the participants are almost matched among the groups. Only the HTN and COV + HTN groups were significantly older than the CTL group *(P* < 0.05). In non-HTN groups, the blood pressure was below the level required for a hypertension diagnosis; meanwhile, the arterial systolic and diastolic blood pressure of the COV (*P* < 0.05), HTN (*P* < 0.001), COV + HTN (*P* < 0.001), COV + DM (*P* < 0.05), and COV + OB (*P* < 0.05) groups were statistically higher than those of the CTL group. As expected, BMI was higher in the OB and COV + OB groups compared to that in the CTL group (*P* < 0.001).

**Table 1:** General characteristics of the study groups

	CTL	HTN	DM	OB	COV	COV + HTN	COV + DM	COV + OB
Number	20	20	18	15	20	18	15	18
Male *n* (%)	10 (50)	9 (46.6)	9 (50)	8 (53.3)	10 (50)	9 (50)	7 (46.7)	10 (55.5)
Age (years)	53.9 ± 3.8	63.5 ± 2.5*	53.5 ± 3.8	48.4 ± 4.3	57.6 ± 3.06	63.2 ± 3.02*	53.5 ± 3.7	49.06 ± 3.2
BMI (kg/m^2^)	21.01 ± 0.48	22.3 ± 0.36	21.2 ± 0.63	31.9 ± 1.29^***^	22.6 ± 0.57	24.2 ± 0.87	25.2 ± 0.79	32.4 ± 1.15^**^
SBP (mmHg)	108 ± 2.3	150 ± 0.2***	115 ± 2.4	116 ± 3.4	123 ± 4.1*	145 ± 4.5***	130 ± 6.08**	124 ± 22*
DBP (mmHg)	68 ± 1.7	89 ± 2.4***	74 ± 1.7	75 ± 1.7	75 ± 2.1	85 ± 3.7**	79 ± 0.3**	82 ± 3.9**

Data are presented in *n* (%) or mean ± SEM. CTL: control (healthy people), COV: COVID-19, HTN: hypertension, DM: diabetes mellitus, OB: obesity, SBP: systolic blood pressure, DBP: diastolic blood pressure, and DABK: des-arg9-bradykinin. * P < 0.05, ** P < 0.01, *** P < 0.001 vs CTL group. *n* = 15–20 in each group.

**Figure 1:**
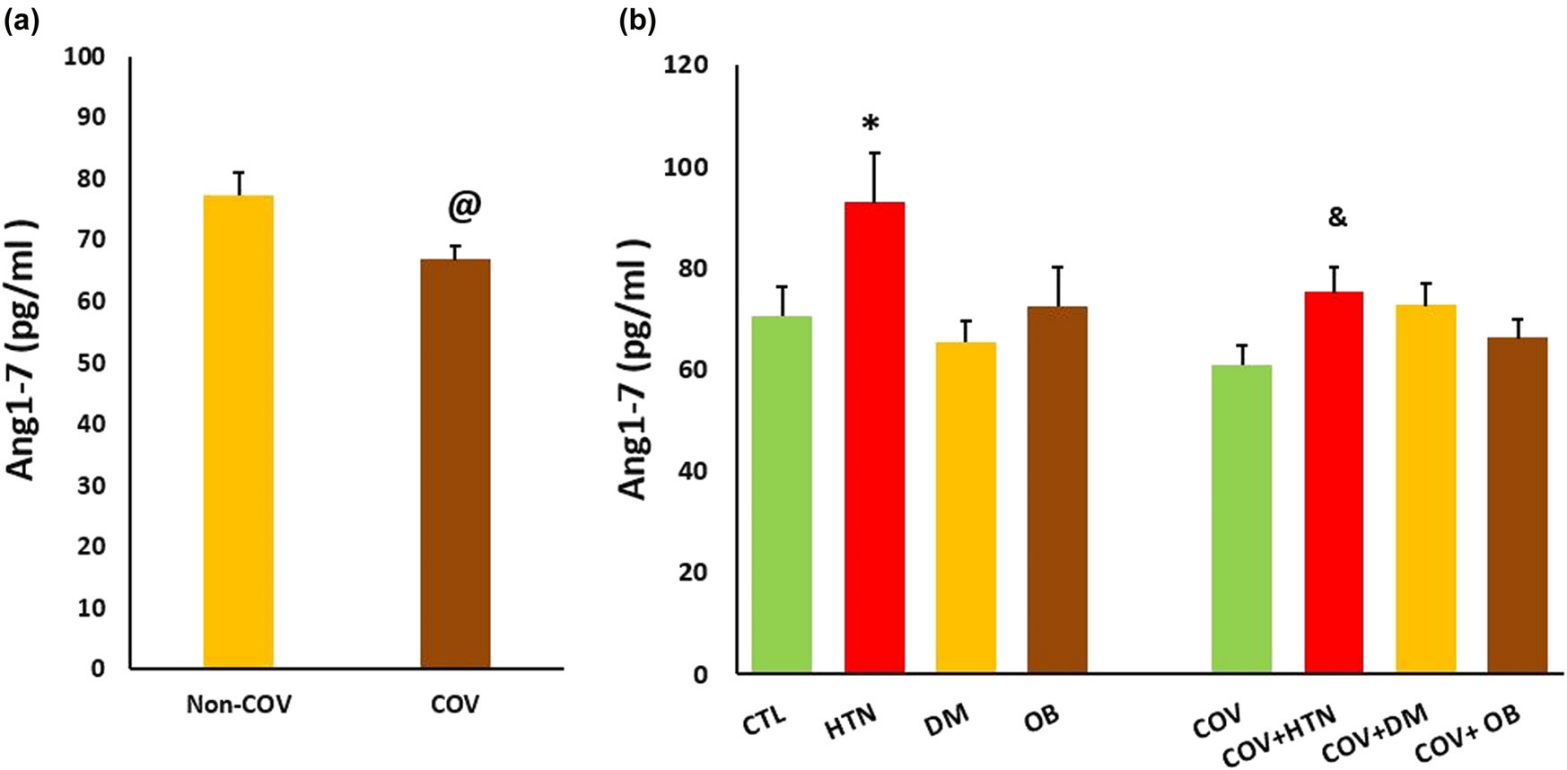
Ang-(1–7) concentration in the study groups: (a) the overall difference between COV (*n* = 69) and non-COV (*n* = 73) participants and (b) the level of ang-(1–7) in COV patients compared to the corresponding non-COV individuals based on the pre-existing risk factors. CTL: control, HTN: hypertension, DM: diabetic mellitus, and OB: obesity. @ *P* < 0.05 vs non-COV, * *P* < 0.05 vs CTL, and & *P* < 0.05 vs HTN group. *n* = 15–20 in each group.

A two-way ANOVA was conducted to examine the interaction effect of COV infection and pre-existing risk factors on the level of ang-(1–7). The study’s findings showed no statistically significant interaction between the effects of COV infection and pre-existing risk factors on ang-(1–7) levels (*P* = 0.08). Simple main effects analysis showed that the pre-existing risk factors significantly affected the levels of ang-(1–7) (*P* < 0.01), and there were differences between COV and non-COV subjects (*P* < 0.05). In patients with hypertension, its level was significantly higher than in the control group (*P* < 0.05). Ang-(1–7) was lower in patients with COV + HTN (*P* < 0.05) compared with the HTN group. In the COV, DM, OB, COV + DM, and COV + OB groups, the ang-(1–7) level did not differ compared to the healthy control group (Figure 1).

**Figure 2:**
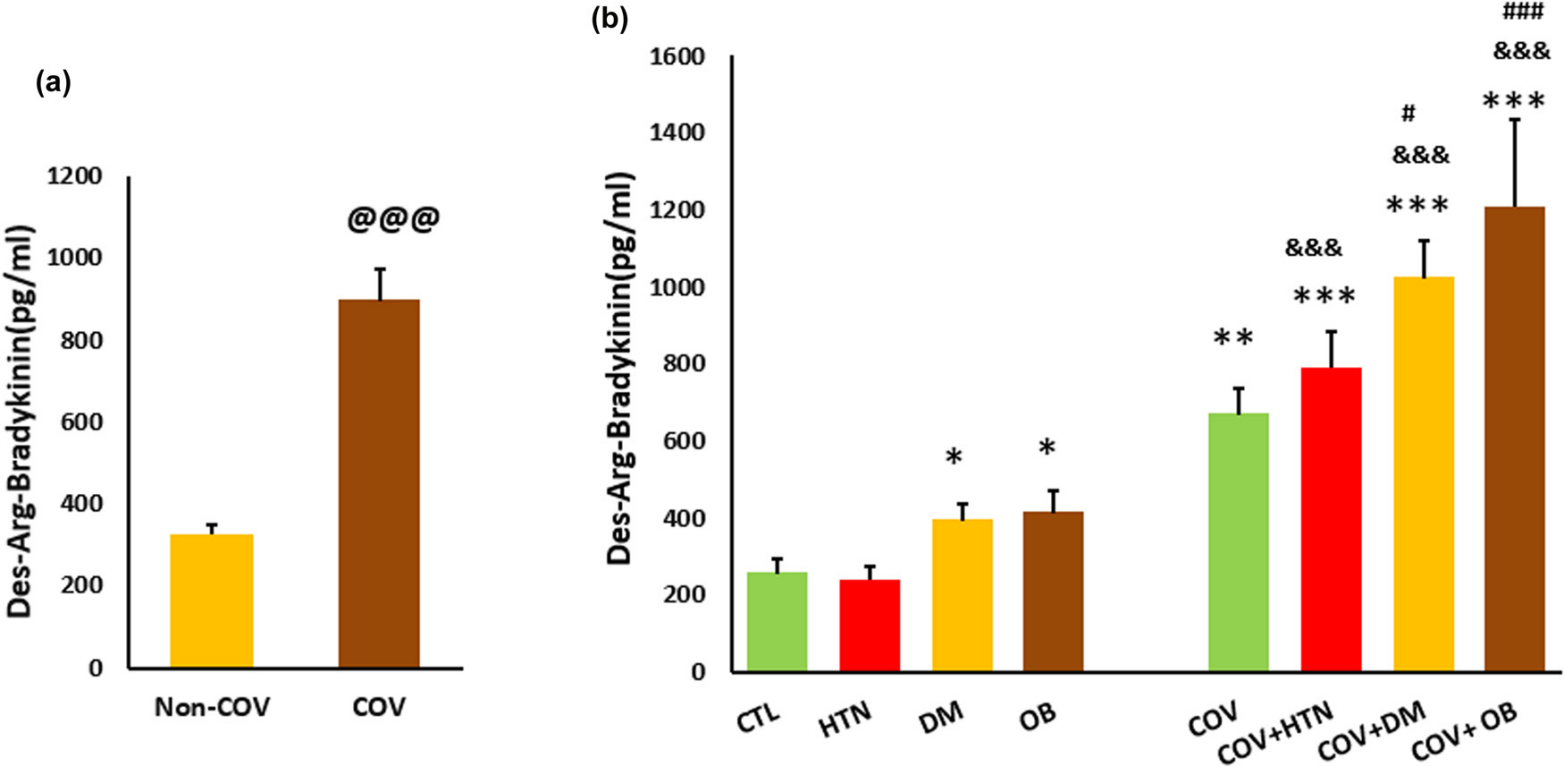
DABK concentration in the study groups: (a) the overall difference between COV (*n* = 69) and non-COV (*n* = 73) participants and (b) the level of DABK in COV patients compared to the corresponding non-COV individuals based on the pre-existing risk factors. CTL: control, HTN: hypertension, DM: diabetic mellitus, and OB: obesity. @@@ *P* < 0.001 vs non-COV, * *P* < 0.05, ** *P* < 0.01, *** *P* < 0.001 vs CTL, &&& *P* < 0.001 vs non-COV corresponding group, # *P* < 0.05 vs DM, ### *P* < 0.001 vs OB group. *n* = 15–20 in each group.

A two-way ANOVA showed a statistically significant interaction between the effects of COV infection and pre-existing risk factors on the levels of DABK (*P* < 0.05). A simple main effect analysis showed that the pre-existing risk factors significantly affected the level of DABK (*P* < 0.001), and the level of DABK was different between COV and non-COV subjects (*P* < 0.001). The concentration of DABK was higher in obese and diabetic subjects without COV than in healthy people (*P* < 0.05). Overall, DABK concentration was higher in COV patients than in non-COV subjects (*P* < 0.001). DABK was higher in control, hypertensive, obese, and diabetic subjects with COV compared with their non-COV counterparts (*P* < 0.001) (Figure 2).

**Figure 3:**
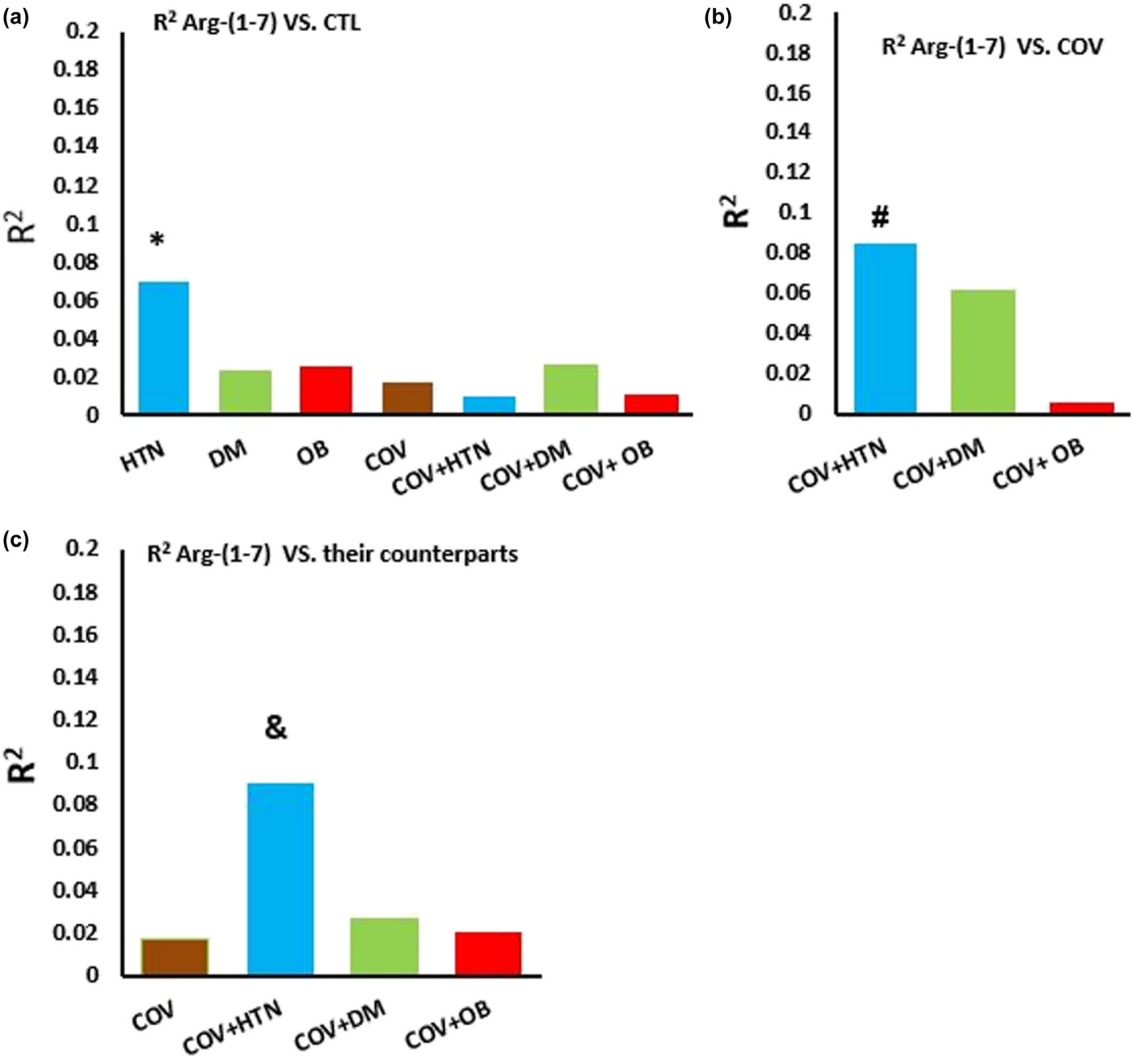
The coefficient of determination (R2; bars) indicates the level of ang-(1–7) relationship in the study groups. Comparing the relationships with the CTL group (a), with COV group (b), and in the COV group with the corresponding non-COV group (c), based on the pre-existing risk factors. CTL: control, HTN: hypertension, DM: diabetes mellitus, and OB: obesity. * *P* < 0.05 vs non-COV (or CTL) group, # *P* < 0.05 vs COV group, & *P* < 0.05 vs counterpart group. *n* = 15–20 in each group.

Linear regression examining the correlation between the level of ang-(1–7) and different risk factors showed that the level of ang-(1–7) positively correlated with hypertension when compared with CTL (*R* ^2^ = 0.07, *P* = 0.04) (Figure 3a). In the COV patients, there was a significant correlation between the ang-(1–7) level and COV + HTN (*R* ^2^ = 0.085, *P* = 0.034) (Figure 3b). The level of ang-(1–7) was significantly lower in the COV + HTN group than in the HTN group (*R* ^2^ = 0.09, *P* = 0.04).

**Figure 4:**
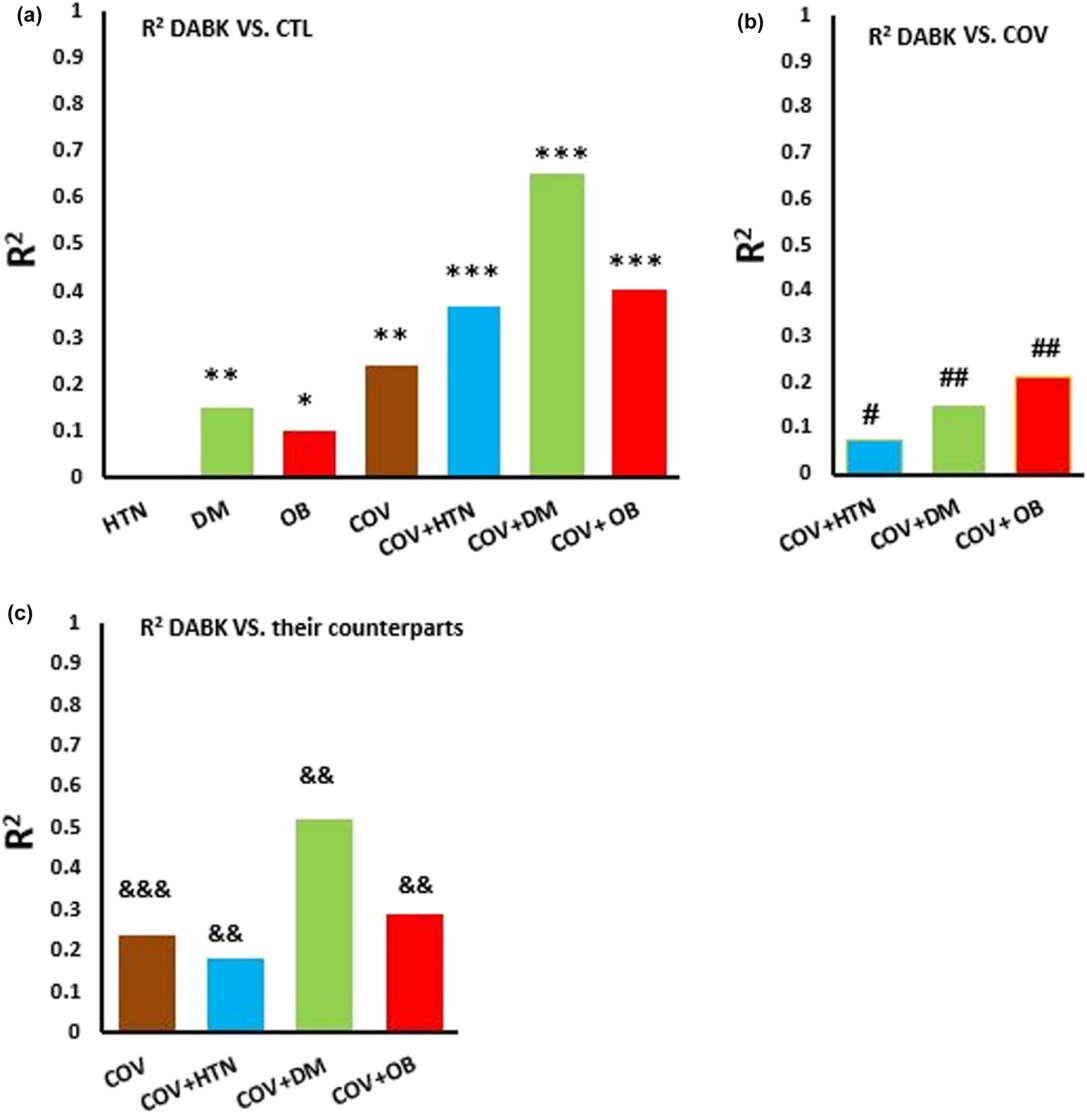
The coefficient of determination (R2; bars) indicates the level of the DABK relationship in the study groups. Comparing with the CTL group (a), with the COV group (b), and the COV group with the corresponding non-COV group (c), based on the pre-existing risk factors. CTL: control, HTN: hypertension, DM: diabetic mellitus, and OB: obesity. * *P* < 0.05, ** *P* < 0.01, *** *P* < 0.001 vs non-COV (or CTL) group, # *P* < 0.05, ## *P* < 0.01 vs the COV group. && *P* < 0.01 and &&& *P* < 0.001 *vs* the counterpart group. *n* = 15–20 in each group.

The level of DABK positively correlated with diabetes (*R* ^2^ = 0.15, *P* = 0.009), obesity (*R* ^2^ = 0.10, *P* = 0.02), COV (*R* ^2^ = 0.24, *P* < 0.001), COV + HTN (*R* ^2^ = 0.37, *P* < 0.001), COV + diabetes (*R* ^2^ = 0.65, *P* < 0.001), and COV + OB (*R* ^2^ = 0.40, *P* < 0.001), when compared with CTL (Figure 4a). In COV patients, there was a significant correlation between the DABK level and COV + HTN (*R* ^2^ = 0.075, *P* = 0.04), COV + DM (*R* ^2^ = 0.15, *P* = 0.01), and COV + OB (*R* ^2^ = 0.21, *P* = 0.003) (Figure 4b). DABK was higher in hypertensive (*R* ^2^ = 0.18, *P* = 0.008), obese (*R* ^2^ = 0.26, *P* = 0.001), and diabetic (*R* ^2^ *=* 0.52, *P* = 0.001) subjects with COV compared with their non-COV counterparts (Figure 4).

A multiple regression analysis was carried out to find the predictors of changes in ang-(1–7) and DABK in serum. The results indicated that the model explains 8% (*R* ^2^ = 0.08, *P* = 0.04) and 9% (*R* ^2^ = 0.09, *P* = 0.01) of the alterations in ang-(1–7) and DABK levels, respectively. The level of DABK is associated with OB (*P* < 0.001), and the level of ang-(1–7) is associated with HTN (*P* < 0.001) (Table 2).

**Table 2:** Multiple linear regression of the level of ang-(1–7) or DABK, as dependent variables, with age, OB, DM, HTN, gender, and serum DABK or ang-(1–7) level

Dependent variable	Predictor	Beta	*P* value
Ang-(1–7)	HTN	18.413	0.001
	Age	0.049	0.749
	OB	3.117	0.583
	DM	0.351	0.948
	Gender	−3.039	0.529
	DABK	−0.003	0.608
DABK	HTN	53.366	0.616
	Age	3.182	0.284
	OB	396.548	0.001
	DM	1.794	0.754
	Gender	9.939	0.284
	Ang-(1–7)	−0.832	0.608

DM: diabetes mellitus, DABK: des-arg9-bradykinin, DM: diabetes mellitus, HTN: hypertension, and OB: obesity.

Up to the end of follow-up, 8.1% of the patients died due to COV. Cox regression indicated that the hazard of death was not dependent on gender, age, DM, OB, and HTN (Table 3).

**Table 3:** Cox proportional Hazard model on the survival of COVID-19 patients with different risk factors (being male and having COV were used as reference)

Variable	Hazard ratio (HR)	95% CI	*P* value
Gender	0.46	0.02–3.34	0.51
Age	0.97	0.78–1.17	0.39
HTN	1.27	0.63–2.49	0.48
DM	1.50	0.70–3.18	0.28
OB	0.98	0.42–2.26	0.96
DABK	1.00	1.0–1.02	0.81
Ang-(1–7)	1.08	0.97–1.012	0.52
COV + HTN vs COV	4.22	0.48–87	0.22
COV + DM vs COV	1.3	0.03–19	0.86
COV + OB vs COV	4.4	0.15–131.7	0.34

DM: diabetes mellitus, DABK: des-arg9-bradykinin, DM: diabetes mellitus, HTN: hypertension, and OB: obesity.

## 4 Discussion

The findings of this study indicated that the level of ang-(1–7) was lower and the level of DABK was higher in COV patients, and the pre-existing risk factors influenced these levels. The level of ang-(1–7) was related to HTN, and the level of DABK was related to obesity.

Due to the critical role of ACE2 in SARS-CoV-2 infection, it is proposed that the altered RAAS and KKS stability may lead, in part, to adverse outcomes and increased risk of mortality in COV patients, especially in individuals with pre-existing comorbidities such as hypertension, diabetes, and obesity [15,28,29].

It was suggested that as a consequence of the binding of the SARS-CoV-2 virus to the membrane ACE2, the availability and activity of ACE2 as well as the level of ang-(1–7) is reduced in COV patients [4]. The reduction of ACE2 has been attributed to the binding of the protein spike of SARS-CoV-2 to soluble ACE2. Consistent with our results, a very recent study indicated that ang-(1–7) levels decrease in COV patients [30]. However, our results highlighted the changes in ang-(1–7) levels of COV patients with underlying risk factors. Although the reduction of ang-(1–7) was low, its reduction was significantly associated with HTN. In contrast, another study has shown that ang-(1–7) levels increase in people with COV [6]. The difference may be due to differences in the stage and severity of the disease. Diversity in ang-2 levels has also been observed, depending on the severity of the viral infection [3].

Ang-(1–7) may be useful in treating the cardiopulmonary outcomes of SARS-CoV-2 owing to its antihypertensive, anti-inflammatory, anti-thrombotic, antiarrhythmic, and vasodilator effects [31]. Animal studies have shown that ang-(1–7) levels decrease in ARDS, and its replacement improves lung function by the reduction of oxidative stress and pulmonary fibrosis [32]. The mechanism of action may be the inhibition of ERK1/2 and NF-kB production [33]. Preclinical findings have indicated that ang-(1–7) improves oxygenation and reduces inflammation in ARDS [9,32]. It was observed that ang-(1–7) alleviated hypertension and diabetes by improvement of endothelial function [34]. Since the endothelium is injured in COV patients, it is likely that the endothelial dysfunction is created by dysregulation of protecting factors such as ang-(1–7).

Our study indicated a higher level of ang-(1–7) in hypertensive subjects, probably due to compensatory mechanisms or the effect of the antihypertensive drugs they take. ACE2 expression increased in hypertensive patients who receive angiotensin receptor blockers (ARB) or ACE1 inhibitors [35].

This study showed that the level of DABK was higher in obese and diabetic subjects compared to the healthy control group. Other studies have also reported an increase in DABK in obese and diabetic adults, which was associated with a higher risk of cardiometabolic diseases [14,36]. Therefore, the elevation of DABK may be responsible for unwanted disorders in COV patients, especially in obesity and diabetes conditions. Based on our results, the level of DABK was significantly associated with obesity. It has been proposed that a “bradykinin storm” is responsible for most of the symptoms of COV, including increased vascular permeability and pulmonary edema [37]. Indeed, bradykinin storms may have a more prominent role than cytokine storms, which have been suggested as the main cause of severe COV symptoms [38]. Overproduction of DABK overactivates B1 receptors and leads to the leakage of pulmonary arteries, edema, and cough [37]. In experimental models of endotoxin-induced pneumonia, loss of ACE2 function leads to the accumulation of DABK, activation of B1 receptors, release of pro-inflammatory chemokines and cytokines, such as TNF-α, from the epithelium, and damage to the lungs [39,40].

We acknowledge the limitations of our study as the number of subjects in each group may be small. This was due to the limitations in finding subjects with only one of the pre-existing risk factors. Second, we measured ang-(1–7) and DABK levels once on the first day of admission of the COV patients and did not follow their changes with the progress in the severity of the disease to have a clearer picture of the correlation between the level of these two variables and the disease progression.

## 5 Conclusion

The level of DABK alters in patients with COV, depending on the pre-existing risk factors such as hypertension, obesity, and diabetes. However, the ang-(1–7) alteration is affected by hypertension. More severe complications in disease symptoms and mortality in patients with these pre-existing risk factors seen in the clinic may be related to alterations of ang-(1–7) and DABK levels. These findings may be useful in devising a more appropriate strategy for treating patients with COV disease.
